# The occurrence of visual and cognitive impairment, and eye diseases in the super-elderly in Japan: a cross-sectional single-center study

**DOI:** 10.1186/s13104-015-1625-7

**Published:** 2015-10-29

**Authors:** Hideki Fukuoka, Masahiro Nagaya, Kenji Toba

**Affiliations:** Division of Ophthalmology, Department of Advanced Medicine, National Center for Geriatrics and Gerontology, 7-430 Morioka-cho, Obu, Aichi 474-8511 Japan; National Center for Geriatrics and Gerontology, Obu, Aichi Japan

**Keywords:** Aged, Aging, Eye disease, Visual impairment, Cognitive impairment, Falls

## Abstract

**Background:**

The current state of eye diseases and treatments in the elderly as well as the relationships between dementia and systemic diseases remain unclear. Therefore, this study evaluated the prevalence of eye diseases, visual impairment, cognitive impairment, and falls (which are an important health issue and are considered one of the Geriatric Giants) in super-elderly people in Japan.

**Methods:**

The subjects were 31 elderly people (62 eyes; mean age: 84.6 ± 8.8 years; age range 61–98 years) who were admitted to a geriatric health services facility. Eye treatment status, systemic diseases, dementia, and recent falls were investigated. Eye examinations including vision and intraocular pressure measurement, and slit-lamp biomicroscopy were conducted.

**Results:**

Mean best corrected visual acuity (logMAR) was 0.51 ± 0.56, and mean intraocular pressure was 13.7 ± 3.5 mmHg. Approximately half of the subjects exhibited excavation of the optic nerve head including cataracts and glaucoma. Ten subjects had visual impairment (i.e., visual acuity of the eye with the better vision <20/40). The mean Hasegawa dementia scale scores between the visually impaired and non-visually impaired groups were 10.2 ± 6 and 16 ± 8 points, respectively (p < 0.05). Furthermore, 70 % of subjects with visual impairment experienced a fall in the past year compared to 48 % of those without visual impairment, although the difference was not significant. Regarding systemic diseases, there were 6, 5, and 15 cases of diabetes, hyperlipidemia, and hypertension, respectively. There was no significant difference between these systemic diseases and visual function after adjusted for age and gender.

**Conclusions:**

The percentages of patients with age-related eye diseases and poor visual acuity in a geriatric health services facility were extremely high. Compared to those without visual impairment, those with visual impairment had lower dementia scores and a higher rate of falls.

## Background

Japan became a super-aged society, more than 21 % of the population aged older than 65, in 2007. Accordingly, improving home health care services for older adults is becoming increasingly important. National policies including the 5 year plan for promotion of measures against Dementia (Orange Plan) have been proposed by the Ministry of Health, Labour, and Welfare [1]. Although there is some information on eye diseases in patients receiving home healthcare, there are no reports related to the current or treatment state of eye diseases among people requiring home healthcare or the relationships between the degree of dementia and systemic diseases. This may be attributable to the lack of attention to home healthcare in the literature, the possibility that home healthcare patients with visual impairment have not received consultation because they cannot easily visit a hospital, or the possibility that large-scale in home vision assessments are currently impractical as the large ophthalmic examination apparatuses required are difficult to bring to patients. Falls are a major problem and a significant cause of mortality due to unintentional injury among the elderly [2]. Therefore, we examine the relationship between eye disease, cognitive impairment, systemic diseases, and falls at a geriatric health service facility that patients attend before returning home or receiving home healthcare.

## Methods

The subjects included 31 elderly people (mean age: 84.6 ± 8.8 years; age range 61–98 years) including 5 men and 26 women who were admitted to a geriatric health services facility between March 2013 and April 2013. The facility is located in Central Japan, which has a mild climate relative to those of Northern and Southern Japan. Furthermore, the facility is 1.6 km from our hospital in order to enable emergency treatment in case the subjects suddenly deteriorated during research. Its capacity is approximately 100 patients, and 37 % of admitted patients returned to home healthcare within 6 months in 2013. All residents were divided into three groups according to the degree of dementia (none, none to mild, moderate and severe dementia) according to the degrees of necessity for nursing care due to dementia. We randomly extracted approximately equal numbers of participants among these groups.

The status of eye treatment in the health services facility was evaluated. Subjects underwent basic ophthalmological examinations by using slit-lamp biomicroscopy (SL-15, Nidek Technologies, Japan) and a monocular indirect ophthalmoscope (NT-300, HEINE Optotechnik, Germany). Corneal and total astigmatism, and refraction were measured by an autorefractor keratometer (ARK-700A, Nidek Technologies, Japan). Intraocular pressure was measured by a non-contact tonometer (NT-3000, Nidek Technologies, Japan). Uncorrected and best corrected visual acuity were measured by a visual acuity system (SC-2000, Nidek Technologies, Japan). Patients with visual acuity <20/40 in the eye with better vision were considered to have visual impairment. The American criteria define visual acuity <20/40 and >20/200 in the eye with better vision as low vision and <20/200 in the eye with the better vision as blind [3].

Systemic diseases such as diabetes, hyperlipidemia, and hypertension were also evaluated. In clinical settings in Japan, the Hasegawa dementia rating scale-revised (HDS-R) and mini-mental state examination (MMSE) Japanese edition are commonly used questionnaires to evaluate cognitive functions. The HDS-R was developed in 1991 and comprises nine questions; the possible score ranges from 0–30, with lower scores indicating more severe dementia. It is well validated in Japan, with 93 % sensitivity and 86 % specificity [4]. Furthermore, the HDS-R is strongly correlated with the MMSE (r = 0.94).

All examinations were conducted by an ophthalmologist using a non-mydriatic fundus camera (Smartscope M5, Optomed, Finland); images were captured simultaneously and subsequently interpreted and judged by glaucoma specialists from an external institution. Depending on the disease severity, the patients who were determined to have a disorder received appropriate emergency treatment or were referred for ophthalmology consultation. All subjects provided informed consent after being briefed about the study. For participants with dementia, we obtained proxy consents from their family members. The study design was approved by the appropriate ethics review boards of the National Center for Geriatrics and Gerontology and relevant geriatric health services facility.

Data are presented as mean ± SD. Statistical analyses were performed with the Statistical Analysis System (SAS, SAS Institute, Cary, NC, USA). The Mann–Whitey U-test and Generalized Linear Model were used for intergroup comparisons. The level of significance in all analyses was set at p < 0.05.

## Results

**Fig. 1:**
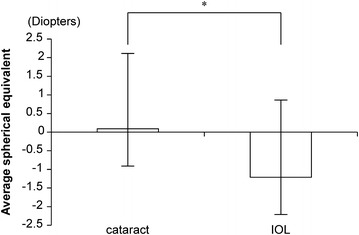
Mean spherical equivalents for cataract eyes and eyes with intraocular lenses (IOL) inserted. The mean spherical equivalents for overall eyes, cataract eyes, and eyes with intraocular lenses inserted were −0.69 ± 2.0, 0.09 ± 2.02, and −1.21 ± 2.07 D, respectively. *p < 0.05

Mean corneal astigmatism was −1.61 ± 1.1 diopters (hereafter, D), with a mean total astigmatism of −1.10 ± 0.48 D (moderate). Regarding the type of astigmatic axis, corneal astigmatism was observed in 32/50 eyes (64 %) with against-the-rule astigmatism and 20/29 eyes (69 %) of those with total astigmatism. The mean spherical equivalents overall, for cataract eyes, and for eyes with intraocular lenses inserted were −0.69 ± 2.0, 0.09 ± 2.02, and −1.21 ± 2.07 D, respectively. Thus, eyes with cataracts were significantly more far-sighted (p < 0.05) (Fig. 1).

Regarding eye diseases of the outer ocular area, 5 eyes had blepharitis (8.1 %) and 1 eye had entropion (1.6 %). Regarding the optic media, 31 eyes had cataracts (50 %) and 31 had intraocular lenses inserted (50 %). Regarding the posterior eye segment, 33 eyes had excavation of the optic nerve head or a cataract (53 %); 2 eyes each had diabetic retinopathy and branch retinal vein occlusion (3.2 % each); 1 eye each was affected with age-related macular degeneration, macular hole retinal detachment, and macular epiretinal membrane (1.6 % each); and 22 had no abnormality (35 %) (Table 1). We observed 45 % (14/31) of participants who have had worse eye with more than one of eye diseases.

**Table 1:** Eye diseases affecting the external eye, intermediate optic media, and posterior segment (*N* = 62)

Part of the eye	Eye disease	*n* (%)
External eye	Blepharitis	5 (8.1)
	Entropion	1 (1.6)
	No abnormality	56 (90.3)
Intermediate optic media	Intraocular lens	31 (50)
	Cataract	31 (50)
Posterior segment	Glaucoma or glaucoma suspect	33 (53)
	Diabetic retinopathy	2 (3.2)
	Retinal vein occlusion	2 (3.2)
	Age-related macular degeneration	1 (1.6)
	Macular hole retinal detachment	1 (1.6)
	Macular epiretinal membrane	1 (1.6)
	No abnormality	22 (35)

**Fig. 2:**
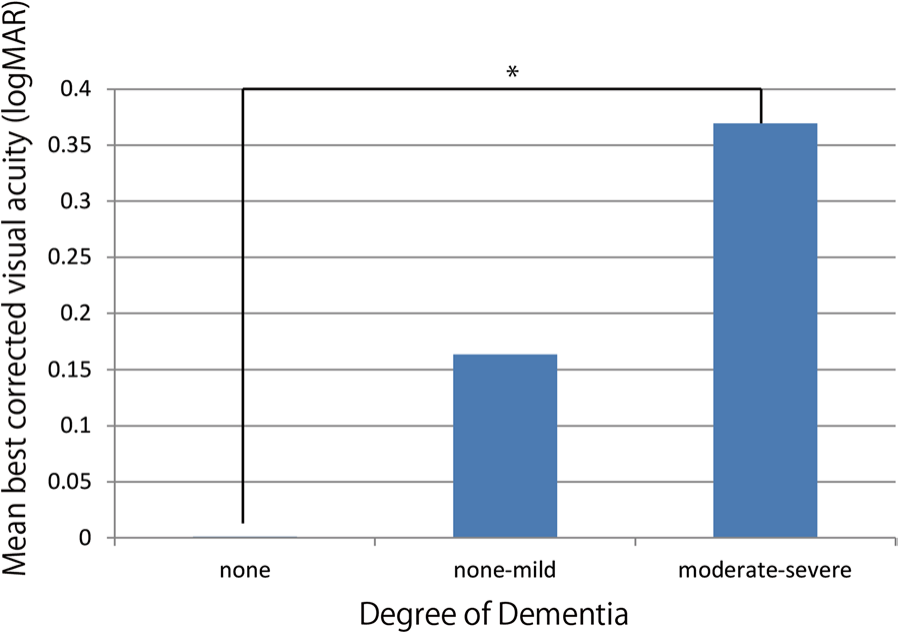
Mean best corrected visual acuity (logMAR) in three groups categorized by degree of dementia (none, none-mild, moderate- severe).*p < 0.05

The mean uncorrected visual acuity (logMAR) and best corrected visual acuity (logMAR) were 0.76 ± 0.56 and 0.51 ± 0.56, respectively, which are insufficient for activities of daily living. Mean intraocular pressure was 13.7 ± 3.5 mmHg. Moreover, 6 subjects (19 %) wore glasses. Regarding vision disorders, 10 subjects had visual impairment (mean age: 85.7 ± 7.8; 25.8 %), including 8 with low vision and 2 with blindness. Thus, the percentages of patients with low vision and blindness were 25.8 and 6.5 %, respectively. We found that there was a significant difference between the three groups categorized by degree of dementia and visual performance after adjusted for age and gender. (p = 0.0092) (Fig. 2).

**Fig. 3:**
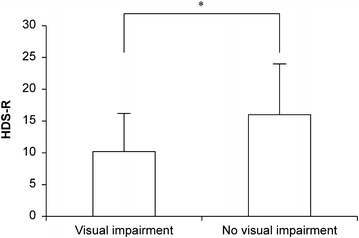
Hasegawa dementia rating scale-revised in subjects with and without visual impairment. *p < 0.05

**Fig. 4:**
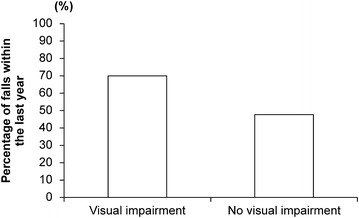
Rates of falling in the past year in subjects with and without visual impairment

We observed 22/31 (71 %) of participants were diagnosed as dementia clinically at the time of admission. The mean HDS-R score was 14 ± 7.8 points. The subjects with visual impairment had a significantly lower HDS-R score than those with no visual impairment (10.2 ± 6 vs. 16 ± 8 points; p = 0.0386; 95 % confidence interval: Fig. 3). Furthermore, more subjects with visual impairment experienced a fall in the past year than those with no visual impairment, although the difference was not significant (70 %, 7/10 cases vs. 48 %, 10/21 cases; p = 0.2802) (Fig. 4). Regarding systemic diseases, there were 6 (19 %), 5 (16 %), and 15 (48 %) cases of diabetes, hyperlipidemia, and of hypertension, respectively (Table 2). In multivariate analysis, we found no significant relationship between these systemic diseases and visual function after adjusted for age and gender (diabetes; p = 0.2267, hypertension; p = 0.9528, hyperlipidemia; p = 0.2176) as well as no significant relationship between these systemic diseases and HDS-R score after adjusted for the same parameters (diabetes; p = 0.3468, hypertension; p = 0.5432, hyperlipidemia; p = 0.3763).

**Table 2:** Systemic diseases (*N* = 31)

Systemic disease	*n* (%)
Hypertension	15 (48)
Diabetes mellitus	6 (19)
Hyperlipidemia	5 (16)

## Discussion

Japan was among the first countries to experience population aging, which began in 1970. Japan became an aged, more than 14 % of the population aged older than 65, and super-aged society in 1995 and 2007, respectively. This coupled with Japan’s declining birth rate has rapidly transformed the population pyramid from stable to contracting. In 2060, the elderly population is predicted to reach 39.9 %, making the further progression of population aging inevitable. Therefore, the Ministry of Health, Labour, and Welfare has pushed forward various policies for the appropriate functional division of healthcare services, enhancing residential and home healthcare services, and the Orange Plan [5], which aims to improve the early detection of dementia and thus early treatment and nursing care to promote home healthcare. Thus, home healthcare is becoming increasingly important. Therefore, we examined the current state of eye disorders in Japan by focusing on the role of geriatric health services facilities.

The corneal and total astigmatism of subjects at a nursing care facility were calculated. Although the proportion of “against-the-rule” astigmatism increases with age [6], the proportions of against-the-rule astigmatism in the present elderly subjects with corneal and total astigmatism were 64 and 69 %, respectively. As such, the proportion of total astigmatism was high, with the mean astigmatism exceeding −1 D. An astigmatic eye is believed to sharpen the retinal image through processing and fine adjustment within the visual cortex. However, the moderate astigmatism among the elderly, which makes this process difficult, can lead to diminished vision if not corrected. In addition, 6 subjects (19 %) wore glasses. Accordingly, some subjects require astigmatism correction but have not received it. The mean spherical equivalent showed that cataract eyes were significantly far-sighted while eyes with intraocular lenses inserted were near-sighted. Possible reasons for this result include the tendency towards far-sightedness with increasing age caused by the opacification of the cortex of the crystal lens [7]. Furthermore, the results may also be affected by the intraocular lenses selected by many practitioners to target postoperative refraction to −1 D.

Diseases affecting the outer ocular area, optic media, and posterior eye segment were evaluated. Regarding diseases of the outer ocular area, blepharitis, which is characterized by the rubefaction of the palpebral portion and attachment of eye mucus to the eyelids and eyelashes, was observed in 8.1 % (5/62) of eyes. In many cases, there was a high degree of eye mucus adhesion, resulting in chronic blepharitis; this is difficult to remove with gauze because of the dryness of the skin area in the palpebral part. Regarding diseases of the optic media, although patients at the present geriatric health services facility receive treatment for cataracts, considering the facility’s distance from the general hospital, approximately half of the subjects had cataracts and intraocular lens insertion, respectively. However, no comorbidities were observed. Many diseases affecting the posterior eye segment were observed. The most common were glaucoma and excavation of the optic nerve head. A Japanese glaucoma epidemiological report shows that the prevalence of glaucoma is 5 % in people aged ≥40 years and 11.4 % in those aged ≥80 years [8]. Furthermore, the morbidity of other systemic diseases and eye disorders is believed to increase with aging. Thus, the high prevalence of systemic diseases and eye disorders among the super-aged in the present study is sufficiently understandable.

The mean uncorrected and best corrected visual acuities were converted into decimal visual acuity, showing extremely poor results of 20/210 and 20/65, respectively. Vision of approximately 20/70 is considered necessary to read a newspaper or TV captions [9]. Thus, the fact that many subjects’ vision fell below this cutoff is important when considering the quality of life and activities of daily living of super-elderly subjects. As of 2005, an estimated 1.45 million (1.1 %) and 190,000 (0.15 %) people in Japan had low vision and blindness, respectively. The percentage of visual impairment at the present facility was very high. Thus, a simple comparison suggests there are many times more low-vision and blind subjects in this population than the general population.

As mentioned above, the HDS-R and MMSE are commonly used to evaluate cognitive functions. However, each scale has its own strengths; for example, the HDS-R is better for assessing Alzheimer’s disease [10]. In clinical actuality, the diagnosis of dementia involves the combination of HDS-R and/or MMSE with other examinations; a questionnaire alone cannot adequately assess the degree of dementia. However, the HDS-R score depends on the stage of dementia. In this study, we observed a significant difference between the three groups categorized by degree of dementia and visual performance after adjusted for age and gender, and the visually impaired group had a significantly lower mean HDS-R score. A possible reason for this is the decline of cognitive function due to visual impairment as inferred from well-established reports analyzing the relationship between visual and cognitive impairment [11].

Elderly people also have an increased risk of falls; accordingly, bone fracture caused by falls is the third-leading cause of becoming bedridden. Risk factors for falling include age [12], a history of falls [13], visual impairment [14], nervous and cardiovascular system disorders [13], pharmacotherapy [15], and gait disorders or postural instability [16]. From an ophthalmologic perspective, hip fracture caused by falls is reported to be associated with vision deterioration, contrast sensitivity, and narrowing of the visual field [17]. Furthermore, a large-scale epidemiological survey shows that a cohort with visual impairment had a higher risk of falls than a cohort without visual impairment [18], suggesting that those with visual impairment may require interventions to prevent falls. In the present study, 70 % (7/10) of subjects with visual impairment experienced a fall within the past year compared to 48 % (10/21) in the non-visually impaired group. However, there was no statistically significant difference in falls between groups.

We found no significant relationship between the three systemic diseases and visual function. We also found no significant relationship between the three systemic diseases and HDS-R scores in multivariable analysis, though this may be a statistical error due to the small sample size (since the three diseases are each known to be a risk factor for dementia [19]).

The miniaturization and advancement of ophthalmological examination apparatuses have greatly enhanced clinical practice; equipment such as autorefractometers, ophthalmic tonometry apparatuses, and eye fundus cameras is now portable. Although there are still some issues faced when providing home healthcare using such equipment, such as the time and human resources required, such service is technically possible.

The present study has some limitations. First, this study was performed at a single geriatric care facility. Future studies should compare the subjects living in geriatric care facilities with an age-matched normal population as a control. However, considering 11 of 31 (35 %) of subjects were ≥90 years old, it is too difficult to collect information from an age-matched normal population. Another limitation is that the HDS-R has one question testing visual memory that requires adequate visual acuity. The response to this question may vary depending on the size of the object presented, thus affecting the score of subjects with visual impairment. In our study, to maximize each participant’s ability to see the object presented, the HDS-R was performed using standardized relatively large objects.

In conclusion, the present results indicate that guiding elderly people with eye disorders to receive screening, diagnosis, and treatment is extremely important in Japan. The results of this study can be used to improve healthcare services for the elderly in Japan.

## References

[1] Nakamura K (2008). A “super-aged” society and the “locomotive syndrome.”. J Orthop Sci.

[2] Department of Trade and Industry (1993). HASS listings for1993, for males and females aged 50 and above for falls.

[3] Congdon N, O’Colmain B, Klaver CC, Klein R, Muñoz B, Friedman DS (2004). Causes and prevalence of visual impairment among adults in the United States. Arch Ophthalmol.

[4] Homma A (1992). Assessment and treatment of patients with dementia of the Alzheimer type. Nihon Ronen Igakkai Zasshi.

[5] Ministry of Health, Labour, and Welfare. Five-year plan for promotion of measures against Dementia (Orange Plan). 2013. http://www.mhlw.go.jp/stf/houdou/2r9852000002j8dhatt/2r9852000002j8ey.pdf. Accessed 16 Aug 2014. (in Japanese).

[6] Asano K, Nomura H, Iwano M, Ando F, Niino N, Shimokata H (2005). Relationship between astigmatism and aging in middle-aged and elderly Japanese. Jpn J Ophthalmol.

[7] Saunders H (1986). A longitudinal study of the age-dependence of human ocular refraction–I. Age-dependent changes in the equivalent sphere. Ophthalmic Physiol Opt.

[8] Iwase A, Suzuki Y, Araie Yamamoto T, Abe H, Shirato S (2004). The prevalence of primary open angle glaucoma in Japanese: the Tajimi Study. Ophthalmology.

[9] American National Standards Institute (ANSI) (1988). American national standard for human factors engineering of visual display terminal workstations. ANSI/HFS Standard No. 100-1988.

[10] Kim KW, Lee DY, Jhoo JH, Youn JC, Suh YJ, Jun YH (2005). Diagnostic accuracy of mini-mental status examination and revised hasegawa dementia scale for Alzheimer’s disease. Dement Geriatr Cogn Disord.

[11] Fukuoka H, Sutu C, Afshari NA. The impact of cataract surgery on cognitive function in an aging population. Curr Opin Ophthalmol. (in press). 10.1097/ICU.0000000000000226 26569525

[12] Campbell AJ, Borrie MJ, Spears GF (1989). Risk factors for falls in a community-based prospective study of people 70 years and older. J Gerontol.

[13] Tinetti ME, Speechley M, Ginter SF (1988). Risk factors for falls among elderly persons living in the community. N Engl J Med.

[14] Coleman AL, Stone K, Ewing SK, Nevitt M, Cummings S, Cauley JA (2004). Higher risk of multiple falls among elderly women who lose visual acuity. Ophthalmology.

[15] Woolcott JC, Richardson KJ, Wiens MO, Patel B, Marin J, Khan KM (2009). Meta-analysis of the impact of 9 medication classes on falls in elderly persons. Ann Intern Med.

[16] Nevitt M, Cummings S, Kidd S, Black D (1989). Risk factors for recurrent non-syncopal falls. JAMA.

[17] Squirrell DM, Kenny J, Mawer N, Gupta M, West J, Currie ZI (2005). Screening for visual impairment in elderly patients with hip fracture: validating a simple bedside test. Eye.

[18] Ivers RQ, Cumming RG, Mitchell P, Attebo K (1998). Visual impairment and falls in older adults: the Blue Mountains Eye Study. J Am Geriatr Soc.

[19] Duron E, Hanon O (2008). Vascular risk factors, cognitve decline, and dementia. Vasc Health Risk Manag.

